# Platelet-Expressed TNFRSF13B (TACI) Predicts Breast Cancer Progression

**DOI:** 10.3389/fonc.2021.642170

**Published:** 2021-03-17

**Authors:** Clemens Hinterleitner, Yanjun Zhou, Claudia Tandler, Jonas S. Heitmann, Korbinian N. Kropp, Martina Hinterleitner, André Koch, Andreas D. Hartkopf, Lars Zender, Helmut R. Salih, Stefanie Maurer

**Affiliations:** ^1^Department of Medical Oncology and Pneumology (Internal Medicine VIII), University Hospital Tuebingen, Tuebingen, Germany; ^2^Cluster of Excellence iFIT (EXC 2180) “Image-Guided and Functionally Instructed Tumor Therapies”, University of Tuebingen, Tuebingen, Germany; ^3^Clinical Collaboration Unit Translational Immunology, German Cancer Consortium (DKTK), Department of Internal Medicine, University Hospital Tuebingen, Tuebingen, Germany; ^4^Department of Hematology, Medical Oncology and Pneumology, University Medical Center of Mainz, Mainz, Germany; ^5^Department of Obstetrics and Gynecology, University Hospital Tuebingen, Tuebingen, Germany; ^6^German Cancer Research Consortium (DKTK), Partner Site Tübingen, German Cancer Research Center (DKFZ), Heidelberg, Germany; ^7^Department of Radiology, Memorial Sloan Kettering Cancer Center, New York, NY, United States

**Keywords:** breast cancer, platelets, biomarker, metastasis, TACI/TNFRSF13B, cancer

## Abstract

Although treatment options in breast cancer have been improved significantly, predictive biomarkers for disease progression and metastasis are still lacking. Recent studies indicate that several TNF Receptor Superfamily members are involved in breast cancer cell proliferation and survival. Interestingly, *TNFRSF13B* (*TACI*) mRNA level were of prognostic relevance in breast cancer patients. In this study we provide evidence for TACI expression on platelets of breast cancer patients. The level of platelet-expressed TACI (pTACI) was significantly increased on platelets derived from breast cancer patients compared to healthy controls. Upon platelet activation, pTACI was downregulated on the platelet surface of healthy donors and breast cancer patients. Of note, inhibition of matrix metalloprotease (MMP) prevented downregulation of pTACI *ex vivo*, indicating that proteolytic cleavage of pTACI is responsible for reduction of pTACI level. Stimulation of pTACI *via* BAFF, BAFF 60-mer or APRIL did not influence platelet activation and function. Remarkably, pTACI was particularly regulated during tumor progression in our breast cancer cohort. TACI expression levels on platelets were correlated with clinical parameters including tumor stage, occurrence of metastasis and tumor cell proliferation (Ki67). In conclusion, our data emphasize the potential use of platelets as a liquid biomarker in breast cancer.

## Introduction

Considerable data confirmed critical involvement of chronic inflammation in tumor initiation and progression (1). A better understanding of the tumor-microenvironment (TME) which among others comprises a variety of immune cells is essential to further improve treatment options and ultimately the outcome of cancer patients. Immune cells exert several functions in the TME *via* membrane bound and, more importantly *via* several soluble mediators (2, 3). Whereas several immune cell-derived factors, including perforin, granzyme, FasL or IFNγ, mainly contribute to the elimination of cancer cells, pro-inflammatory cytokines like TGF-ß, IL-6, IL-8 can promote tumor growth, angiogenesis, tumor immune evasion and epithelial-to-mesenchymal transition (EMT) (4–8). In addition, tumor cells can adopt immunomodulatory signaling pathways to promote cell growth and survival.

Besides contribution to B cell activation, proliferation and plasma cell differentiation, autocrine secretion of tumor necrosis factor (TNF) superfamily (TNFSF) members BAFF (TNFSF13B) and APRIL (TNFSF13) have been described to prevent B chronic lymphocytic leukemia (B-CLL) cells from apoptosis (9). In breast cancer several TNF receptor superfamily (TNFRSF) members BAFF, APRIL, BCMA (TNFRSF17) and TACI (TNFRSF13B) have been associated with tumor initiation and progression (10–13).

Platelets interact with both, tumor cells and immune cells and thereby play a prominent role within the TME (14). They promote tumor growth *via* the supply of growth factors/chemokines, EMT and tumor immune evasion (15–18). We and others demonstrated that tumor-educated platelets (TEP) can express a manifold of tumor necrosis factor superfamily members including LIGHT (TNFSF14), GITRL (TNFSF18), OX40L some of which are known immune checkpoint molecules (17, 19, 20). Whereas platelet-derived GITRL was shown to suppress reactivity of NK cells (20), the immunoregulatory role of several other platelet-derived molecules is so far unknown.

We here comprehensively study the expression of the TACI (transmembrane activator and CAML interactor, TNFRSF13B) on platelets of breast cancer patients. Interestingly, we found that pTACI is regulated during platelet activation *via* at least one MMP. Moreover, platelet-derived TACI (pTACI) was found to be specifically regulated during breast cancer progression and metastasis. Of note, pTACI expression was shown to be of prognostic relevance for the occurrence of metastasis and might thus serve as novel, platelet-based, biomarker suitable for liquid biopsies in breast cancer.

## Material and Methods

### Reagents

Paraformaldehyde was purchased from Affymetrix (Santa Clara, CA). Anti-human TACI antibody (clone 165604) and the respective isotype control were from R&D Systems (Minneapolis, MN). CD41a-PeCy5, CD61-FITC and CD62P-FITC were from BD Pharmingen (San Diego, CA). The goat anti-mouse PE conjugate was from Dako (Glostrup, Denmark). Bicoll Separating Solution was purchased from Biochrom AG (Berlin, Germany). Recombinant human BAFF (rhBAFF) and recombinant human APRIL (rhAPRIL) was from PeproTech (Rocky Hill, NJ, USA). Thrombin Receptor Activator Peptide 6 (TRAP-6), collagen and ADP was purchased from SigmaAldrich (St. Louis, MO). Citrate buffer contained 10 mM sodium citrate, 150 mM NaCl, 1 mM EDTA, 1% dextrose, pH 7.4. GI254023 was from Tocris (Bristol, UK) and Batimastat was from Calbiochem (Darmstadt, Germany).

### Patients

During 2019-2020, blood samples from 70 breast cancer patients treated at the Department of Obstetrics and Gynecology and the Department of Medical Oncology and Pneumology were included in our prospective study. The study was approved by IRB (ethics committee of the Faculty of Medicine of the Eberhard Karls Universitaet Tuebingen) and of the University Hospital (13/2007V). Written informed consent in accordance with the Helsinki protocol was given in all cases. The patient characteristics in detail are given in **Table 1**.

**Table 1:** Patient characteristics of the breast cancer cohort.

Patient characteristics	Total
	(n = 70)
**Gender**	
female sex, n (%)	69 (98.6)
**Age**	
Age in years, mean–yr. ± SD	60.3 ± 11.4
(range)	(27 to 87)
**TNM classification, n (%)**	
Stage	
T0	5 (7.1)
T1	22 (31.4)
T2	26 (37.1)
T3	11 (15.7)
T4	6 (8.6)
Node	
N0	43 (61.4)
N1	15 (21.4)
N2	7 (10)
N3	5 (7.1)
Metastasis	
M0	53 (75.7)
M1	17 (24.3)
**Localization of primary tumor**	
Right	22 (31.4)
Left	48 (68.6)
**Histological grading, n (%)**	
G1	5 (7.1)
G2	36 (51.4)
G3	28 (40)
Unknown	1 (1.4)
**ER positive, n (%)**	57 (81.4)
**PR positive, n (%)**	43 (61.4)
**Her2 postive, n (%)**	13 (18.6)
**Treatment, n (%)**	
Chemotherapy, n, (%)	33 (47.1)
Endocrine based therapy, n (%)	17 (24.3)
HER2-targeted therapy, n (%)	13 (18.6)
**Therapy line at the time-point of blood sampling**	
Adjuvant, n (%)	20 (28.6)
1st line, n (%)	16 (22.9)
2nd line, n (%)	8 (11.4)
>2nd line, n (%)	7 (10)

n, number; T, tumor; N, lymph node; M, metastasis; %, Percentage; yr., years; SD, Standard deviation; G, grading; ER, estrogen receptor; PR, progesterone receptor.

### Preparation of Platelets

Preparation of platelets was performed as previously described (17).

### Immunofluorescence

For immunofluorescence analysis, platelets of breast cancer patients and healthy donors (HD) were fixed in 4% PFA in PBS (10 min at 4°C). Platelets were blocked using a BSA blocking solution containing 5% BSA, 0.2% Triton X-100, 0.1% Tween for 60 minutes. As primary antibody anti-TACI (1:200, R&D Minneapolis, MN) and anti-CD61 (1:500, ThermoFisher, St. Louis, MO) were used; as secondary antibodies Alexa-Fluor 594 labelled anti-rabbit (1:1000, Invitrogen, Carlsbad, CA) and Fluor 488 labelled anti-mouse (1:1000, Invitrogen) were used. Slides were mounted in fluorescent mounting medium. Pictures were acquired using an Olympus BX63 microscope and a DP80 camera (Olympus, Shinjuku, Japan). Quantification of platelet size and fluorescence intensity was performed using an ImageJ script (v.1.52).

### Flow Cytometry

Flow cytometry was performed using saturating concentrations of fluorescence-conjugates or unlabeled antibodies followed by a goat anti-mouse PE conjugate (1:100) as secondary antibody. Analysis was performed using a FACS Canto or a FACS Fortessa (BD Biosciences, Heidelberg, Germany). Percent positive cells were calculated as follows: “percent surface expression obtained with specific antibody” − “percent surface expression obtained with isotype control”. Platelets were selected by CD41a+ and CD62P− (resting) or CD41+ and CD62P+ (activated).

### Static Platelet Adhesion and Aggregation Assay

Wells with an inner diameter of 11 mm were treated with poly-L-Lysin (2 µg/cm²) in the presence of either rhBAFF (50, 100, 500 ng/mL) or collagen (10 µM) for 120 min at 37°C followed by washing with PBS (3x). Platelets (8x10^7^/mL) were added to the rhBAFF-coated or collagen-coated wells and incubated for 30 min at 37°C. After washing with citrate buffer, platelets were fixed with 4% PFA for 10 min at -20°C. Wells were imaged using a Olympus BX63 microscope and a DP80 camera (Olympus, Shinjuku, Japan). Quantitative analysis was performed using an ImageJ script (v.1.52). Platelet aggregation was analyzed using the 4-channel light transmission platelet aggregometer APACT 4004 (Elitech, Puteaux, France) according to the manufactures instructions. ADP (10 µM), TRAP-6 (10 µM), rhBAFF (500 ng/mL) and rhAPRIL (500 ng/mL) were used as platelet agonists.

### The Cancer Genome Atlas Database (TCGA) Analysis

Analysis of different TNFSF gene expression profiles were performed in the TCGA dataset as recently described (21). Briefly, Kaplan-Meier survival estimations from a total of 1,006 TCGA breast cancer samples were analyzed based on their expression of *BCMA*, *BAFF-R* and *TACI*. Median TNFSF mRNA expression levels were used to group the patients. Differences in the survival outcomes were calculated using log-rank p-value.

### Statistics

For continuous variables student’s t test, Mann-Whitney U test or one-way ANOVA was used. For categorical data we used chi‐squared test or Fisher’s exact test. Correlation of platelet activation and pTACI expression, intensity ratio (TACI/CD61) and platelet area, as well as Ki67 level and pTACI expression was analyzed using simple linear regression analysis. The predictive value of pTACI was evaluated by examining the area under the receiver‐operator characteristic (ROC) with a confidence interval of 95%. For correlation studies of pTACI and different clinical parameters Odds ratios (OR) were calculated. High pTACI expression was defined as follows: pTACI high = mean pTACI (HD) + 2SD pTACI (HD). We investigated the prognostic value of several mRNA levels in human breast cancer in the TCGA dataset using www.oncolnc.org (21). Additional analysis of preliminary biomarkers was performed using microarray data of 1,809 breast cancer patients (www.kmplot.com) (22). Overall survival (OS) was calculated using the Kaplan‐Meier method. Hazard ratios (HR) were determined using Cox regression analysis. All statistical tests were considered significant when p was below 0.05.

## Results

### Expression of Tumor Necrosis Factor Superfamily Members BCMA, BAFF-R, and TACI mRNA on Breast Cancer Tissue and Platelets of Breast Cancer Patients

**Figure 1:**
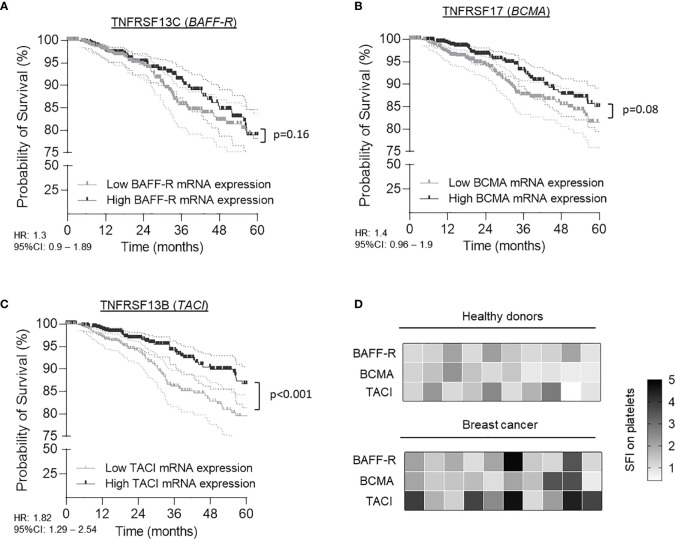
Expression of BAFF-R (TNFRSF13C), BCMA (TNFRSF17) and TACI (TNFRSF13B) in breast cancer patients and association with survival. **(A–C)** Association of *BAFF-R* **(A)**, *BCMA* **(B)** and *TACI* **(C)** mRNA expression with patient survival was calculated using the TCGA Research Network database. Gene expression was analyzed from tumor tissue of 1,006 patients. Low and high expression was defined according to the respective median mRNA levels of the entire cohort. **(D)** Specific fluorescence intensity (SFI, defined as fold change ‘specific signal obtained by specific antibody’/’signal obtained by respective control’) of BAFF-R, BCMA and TACI surface expression of platelets from 10 HD and 10 breast cancer patients were determined by flow cytometry.

To gain a better understanding of TNFRSF members in tumor progression and in particular to extend recent work in breast cancer (13), we comprehensively analyzed available data on mRNA levels of *BAFF-R*, *BCMA* and *TACI* in a collective of 1,006 TCGA breast cancer patients. In contrast to *BAFF-R* (p=0.16, HR: 1.3 95%CI: 0.9-1.89) and *BCMA* (p=0.08, HR: 1.4 95%CI: 0.96-1.9) mRNA level, high *TACI* mRNA level were associated with a significant overall-survival (OS) benefit (p<0.0.1, HR: 1.82, 95%CI: 1.29-2.54) (**Figures 1A–C**). The median survival in the group of patients with high *TACI* level was 148.5 months (5-year survival rate: 85.9%) as compared to 133.1 months (5-year survival rate: 79.5%) in the cohort of patients with low TACI mRNA level. Human TACI is alternatively spliced, thus in breast cancer two isoforms are found (short: uc002gqt.1, coding exon count: 4 and long: uc002gqs.1, coding exon count: 5) which display one and two ligand binding domains, respectively, and may alter in receptor functionality (23, 24). While the short isoform was predominantly found in the TCGA cohort (75% of all patients), it is not known whether and how these variants are associated with tumor progression and clinical outcome in breast cancer (**Supplementary Figure 1**).

It has been described that platelets and tumor cells can communicate *via* an active exchange of tumor- and platelet-specific molecules (20, 25). To investigate the role of platelets in our particular context we analyzed the platelet surface expression of the TNFRSF members BAFF-R, BCMA and TACI in a screening cohort of 10 healthy donors (HD) and 10 breast cancer patients using flow cytometry. Platelets were identified as CD41a-positive subcellular fragments. As shown in **Figure 1D**, we observed a substantial inter-individual variability in the protein surface expression of all three TNFRSF members in HD and breast cancer patients. However, breast cancer patients displayed significant elevated specific fluorescence intensity (SFI) levels of platelet-derived TACI (pTACI) compared to HD (p=0.02). In contrast, no differences between both groups were observed regarding BAFF-R (p=0.11) and BCMA (p=0.17) and expression on platelets.

### Characterization of pTACI in Healthy Donors and Breast Cancer Patients

**Figure 2:**
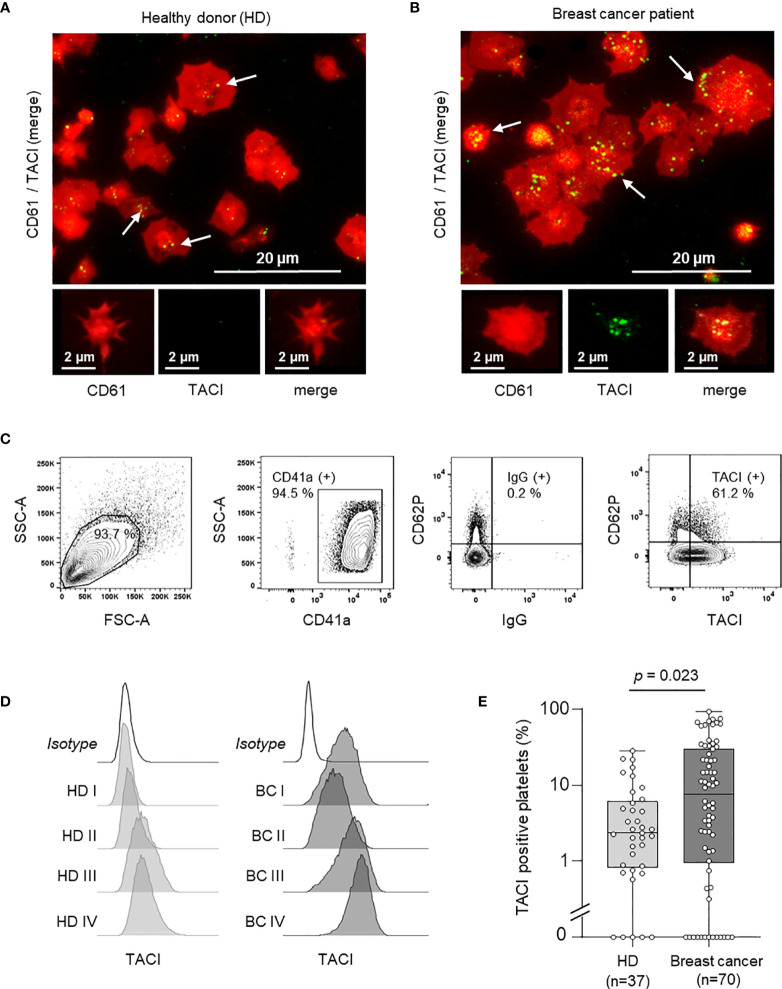
Expression of platelet-derived TACI (pTACI). **(A, B)** Immunofluorescence analysis of pTACI (green) and CD61 (red) expression in platelets from representative HD. White arrows highlight TACI expression in HD and breast cancer patients. **(A)** and breast cancer patient **(B)**. **(C)** Gating strategy used to analyze platelets *ex vivo*. **(D)** Surface pTACI level of platelets derived from HD and breast cancer patients were determined by flow cytometry. Representative results obtained from HD and breast cancer patients are shown. **(E)** The percentage of pTACI positive platelets in 37 HD and 70 breast cancer patients is shown. Within each box, horizontal lines denote 25^th^ percentile, median, 75^th^ percentile (bottom to top) of each group’s distribution values. Whiskers indicate maximal and minimal expression levels (p = 0.023).

Since a relevant increase in pTACI expression was observed in our screening cohort (**Figure 1D**), we further validated these finding using immunofluorescence analysis of platelets from HD and breast cancer patients (**Figures 2A, B**). Platelets were identified *via* CD61 (also referred to as GPIIIa or integrin β3). Compared to platelets obtained from HD (**Figure 2A**), pTACI levels were enhanced in platelets derived from breast cancer patients (**Figure 2B**). To further confirm this observation, we analyzed the amount of TACI positive platelets in a cohort of 37 HD and 70 breast cancer patients using flow cytometry (**Figures 2C–E**). TACI expression was analyzed on CD41+ subcellular fragments in platelet rich plasma (**Figure 2C**) and representative results of pTACI levels obtained in both cohorts are shown (**Figure 2D**). Again, we observed a pronounced inter-individual variability in both cohorts. Nevertheless, pTACI expression was significantly increased in platelets derived from breast cancer patients (HD: 2.34%, 95%CI: 0.1 – 28.16% vs. breast cancer: 23.84%, 95%CI: 0-71.36%, p=0.023) (**Figure 2E**).

### Correlation of pTACI Expression and Platelet Activation

**Figure 3:**
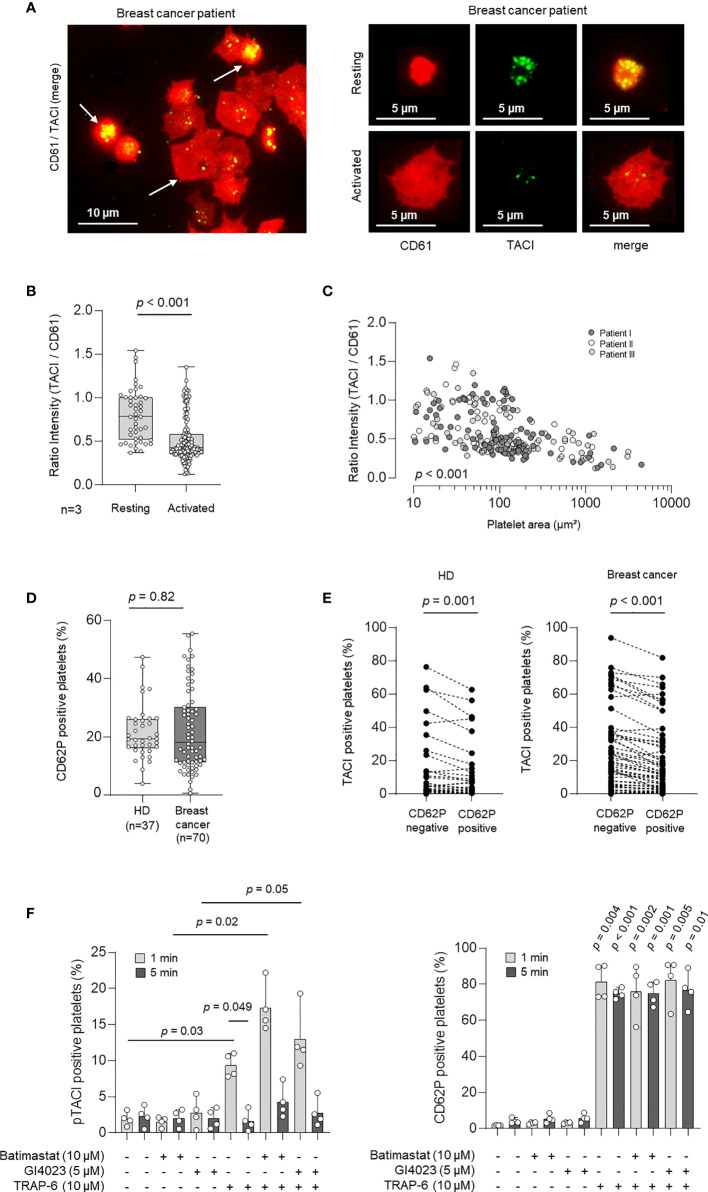
Expression and regulation of pTACI upon platelet activation. **(A)** Immunofluorescence analysis of pTACI (labeled in green) and CD61 (labeled in red) expression in activated and resting platelets from a breast cancer patient. **(B)** Ratio intensity of TACI/CD61 in resting and activated platelets obtained from 3 breast cancer patients (p < 0.001). Dots indicate a single quantified platelet. **(C)** Correlation of the ratio intensity of TACI/CD61 and the platelet area (µm²), (p < 0.001). Each dot indicates a single quantified platelet area (Data from patient I are labeled in dark gray, data from patient II in white, data from patient III in light gray). **(D)** Expression of the platelet activation marker CD62P (P-selectin) in platelets from HD and breast cancer patients *ex vivo (*p = 0.082*)*. **(B, D)** Within each box, horizontal lines denote 25^th^ percentile, median, 75^th^ percentile (bottom to top) of each group’s distribution values. Whiskers indicate maximal and minimal expression levels. **(E)** pTACI level in HD (left panel) and breast cancer patients (right panel) with regard to CD62P expression is shown. **(F)** Expression of pTACI (left panel) and the platelet activation marker CD62P (P-selectin, right panel) in platelets from HD in the presence or absence of TRAP-6, or the indicated metalloproteinase inhibitor (GI254023, ADAM 10; Batimastat, broad spectrum). Platelets were activated for 1 min or 5 min as previously described (20).

It is known that the expression of immunomodulatory ligands and receptors including TNFSF/TNFRSF members is strictly regulated in most cell types (13, 26). However, very little is known about the regulation of these molecules on platelets, especially in the pathophysiologic context of tumor initiation and progression. Since platelet activation is known to be strongly associated with protein expression changes on the platelet surfaces, we next studied the association of pTACI surface level and platelet activation. In a first step, we analyzed pTACI expression and platelet activation *via* immunofluorescence. To this end, morphological changes during platelet activation were assessed and correlated with the size of the respective platelet (**Figure 3A**). Median platelet size in resting platelets was 12.4 µm² (95%CI: 9.7 - 21.2 µm²). Activated platelets were classified based upon their morphology. Based on this, activated platelets showed at least 1.5 fold increased platelet size compared to resting platelets. In a second step, we quantified pTACI fluorescence intensity in all platelets. To quantify the relative pTACI expression in each platelet we calculated the intensity ratio of pTACI/CD61. As shown in **Figure 3B**, the intensity ratio of pTACI/CD61 in resting platelets was significantly higher compared to the ratio obtained in activated platelets (resting: 0.78, 95%CI: 0.39 – 1.45 vs. activated: 0.43, 95%CI: 0.22 – 1.1, p<0.001). In addition, we observed a negative correlation of ratio intensity (TACI/CD61) and platelet covered area/µm² (p<0.001, **Figure 3C**). Based on these findings, activated platelets appear to express less pTACI compared to resting platelets.

To further confirm this finding, we determined the preexisting activation level (CD62P expression) of platelets from HD and breast cancer patients *ex vivo*. We obtained 19.4% of CD62P-positive platelets (95%CI: 10.4-38.6) in HD and 18.1% (95%CI: 5.6-48.7) in breast cancer patients and thus similar platelet activation levels in both groups (*p* = 0.82, **Figure 3D**). Next, we studied pTACI level with regard to CD62P expression in the HD and breast cancer cohorts. Of note, in both groups pTACI expression was significantly enhanced in the resting (CD62P-negative) platelet fraction (HD: p = 0.001, breast cancer: p<0.001, **Figure 3E**). Interestingly, in HD and breast cancer patients the fraction of CD62P positive (activated) platelets was significantly associated with the extent of TACI surface expression change (Δ TACI). Moreover, TACI surface expression change (Δ TACI) was negatively correlated with the pTACI expression in the resting platelet fraction (**Supplementary Figure 2**), indicating that resting platelets with a high pTACI expression show a higher capacity to downregulate pTACI upon activation.

TACI has been found to be proteolytically shed from the surface of activated B cells by the matrix metalloproteinase “a disintegrin and metalloproteinase” 10 (ADAM10) (27). Since ADAM10 is expressed by platelets (28, 29), we set out to assess whether the latter is involved in regulating pTACI surface level. Basal expression of pTACI on resting platelets was not altered in the presence of the broad spectrum matrix metalloproteinase inhibitor Batimastat or the ADAM10 inhibitor GI254023 (**Figure 3F**, left panel). pTACI level were upregulated upon activation with the platelet superagonist TRAP-6 (p=0.03). This effect was much more pronounced in the presence of Batimastat (p = 0.02). The same trend was seen with the ADAM10 inhibitor although not reaching statistical significance in this experimental setting (p=0.05). Together, these data indicate that platelet-derived matrix metalloproteases, and likely ADAM10, are involved in the proteolytic cleavage of pTACI from the platelet surface following platelet activation. Notably, CD62P expression was not altered in the presence of either of the inhibitors (**Figure 3F**, right panel) but as expected in the presence of TRAP-6 (all p<0.05).

### Functional Characterization of rhBAFF and rhAPRIL on pTACI

**Figure 4:**
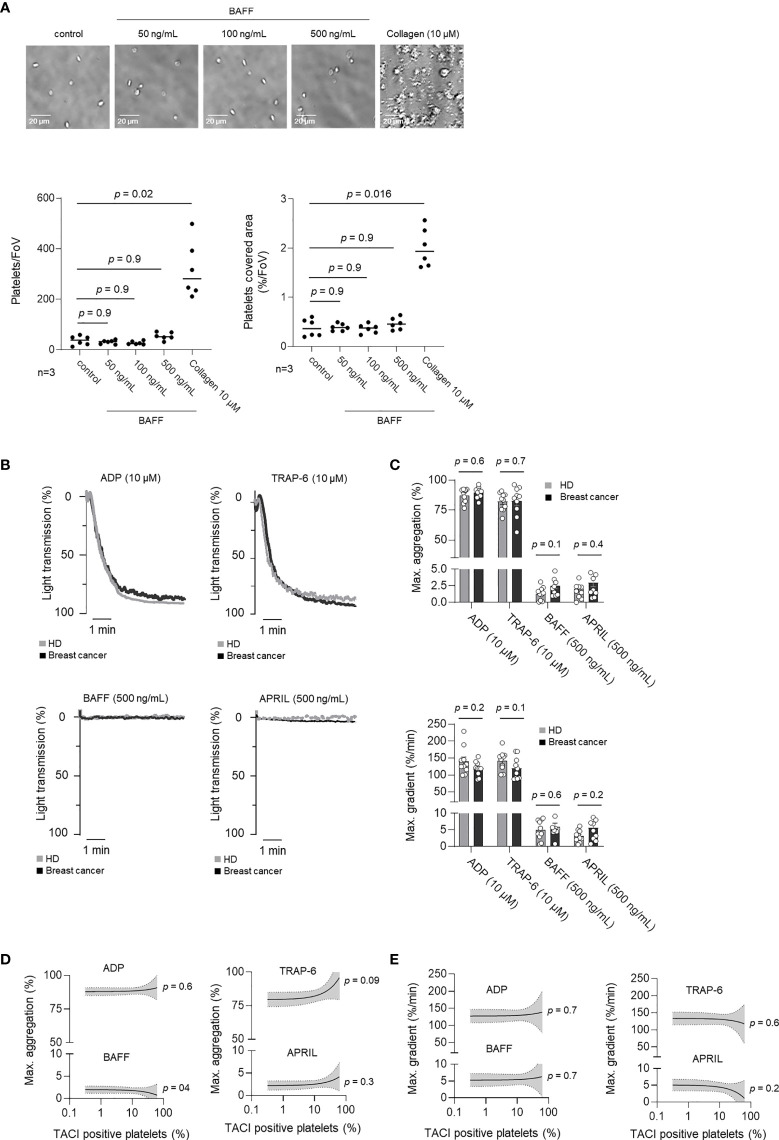
Influence of BAFF and APRIL on pTACI on platelet activation. **(A)** Representative images of platelet adhesion in the presence or absence of rhBAFF (50-500 ng/mL) or collagen (10 µM). The lower left panel displays the quantitative analysis of the platelet adhesion assay per field of view (FoV). The lower right panel shows the quantitative analysis of platelet covered area per field of view (%/FoV). **(B, C)** Platelet aggregation was studied in the presence or absence of classical platelet agonists ADP, TRAP-6 (upper panel) or TACI ligands BAFF, APRIL (lower panel). Percentage of light transmission during the indicated time interval **(B)** and maximum aggregation levels or gradient **(C)** are shown. Dots indicate data obtained with individual HD (gray) and breast cancer patients (black) (n =10 each). The median value obtained from all donors within one group is indicated. **(D, E)** Correlation of maximum aggregation **(D)** or maximal gradient **(E)** and specific pTACI expression. Line indicates linear regression, gray area 95%CI interval.

TNFSF/TNFRSF dependent signaling plays a prominent role in both, non-malignant and malignant cells (13, 26) However, the role of TNFSF/TNFRSF in platelets is largely unknown. Accordingly, we analyzed whether rhBAFF or rhAPRIL induced measurable responses in platelet function. Platelet adhesion was analyzed in the presence of increasing concentrations of rhBAFF (50, 100, 500 ng/mL) or the classical platelet agonists collagen (10 µM), which served as a positive control. In contrast to collagen, rhBAFF did not affect platelet adhesion under static conditions (**Figure 4A**). To further evaluate the putative relevance of rhBAFF and rhAPRIL on platelet aggregation under shear stress, we performed platelet aggregation studies additionally. In contrast to ADP (10 µM) and TRAP-6 (10 µM), neither rhBAFF nor rhAPRIL substantially induced platelet aggregation (**Figures 4B, C**). In addition, maximal aggregation (**Figure 4D**) and maximal gradient (**Figure 4E**) after stimulation with TRAP-6, ADP, rhBAFF or rhAPRIL were not correlated with pTACI expression. The oligomerization state critically determines functionality of many TNFSF, and it has been shown that TACI in particular is only activated by oligomerized BAFF or APRIL (30). We thus performed the aforementioned analyses not only with low oligomeric rhBAFF but also in the presence of a BAFF 60-mer, which confirmed that pTACI stimulation by its highly oligomerized ligand BAFF did not alter platelet adhesion or aggregation *in vitro* (**Supplementary Figure 3**). Since it has been described that in B-cells TACI cooperates with TLRs for the induction of immunoglobulin class switch (31) we additionally performed analysis of platelet adhesion to collagen and platelet aggregation to ADP in the presence of BAFF 60-mer. However, in our *ex vivo* experiments BAFF 60-mer did neither influence platelet adhesion to collagen nor aggregation in the presence of ADP (**Supplementary Figure 4**). As a result, stimulation of pTACI alone seems not to be critical for platelet adhesion or aggregation *in vitro*. Further studies are warranted to assess more in detail whether pTACI *in vivo* may synergize with other receptors in mediating platelet functionality.

### Association of pTACI With Clinical Parameters in Breast Cancer

**Figure 5:**
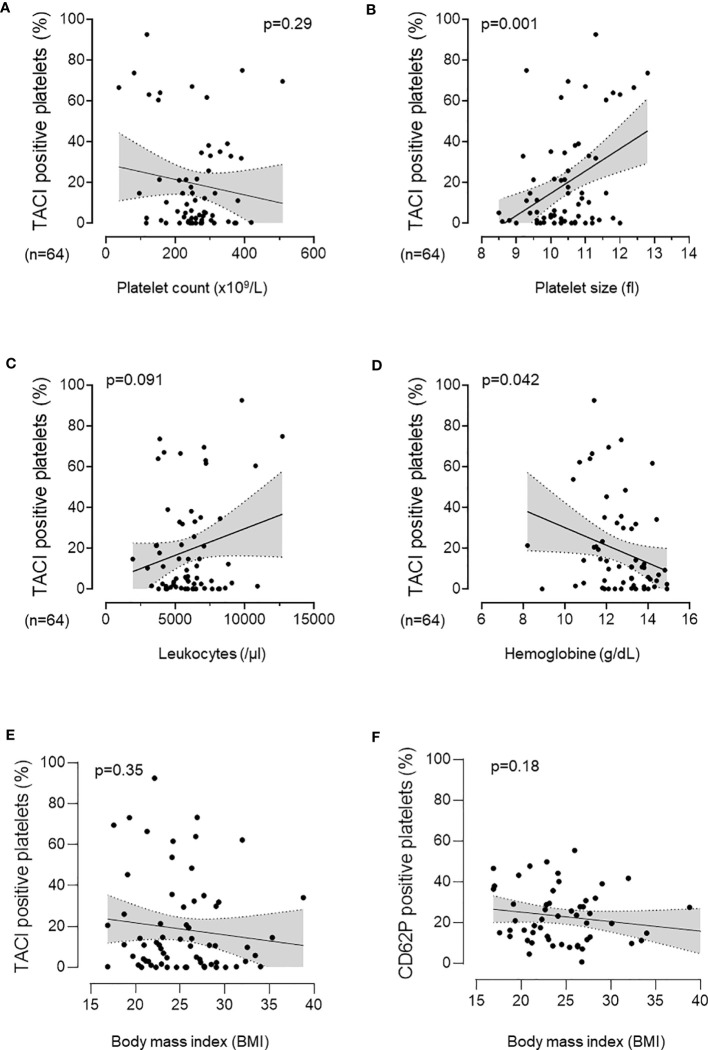
Association of pTACI with hematological and clinical parameters. Correlation of pTACI expression and platelet count (p = 0.29) **(A)**, platelet size (p = 0.001) **(B)**, leukocyte count (p = 0.091) **(C)** and hemoglobin concentration (p = 0.042) **(D)**. Association of pTACI level (p = 0.35) **(E)** and platelet activation (CD62P expression) **(F)** (p = 0.18) with Body mass index (BMI). Line indicates linear regression, gray area 95%CI interval (n = 64 breast cancer patients).

To investigate the role of pTACI in a clinical perspective, we investigated the association of pTACI with several hematological parameters in our cohort of breast cancer patients. Whereas the fraction of TACI positive platelets tended to be negatively associated with the platelet count (p=0.29, **Figure 5A**), a significant correlation of pTACI and the platelet size (p=0.001, **Figure 5B**) was observed. The TACI-positive platelet fraction tended to show a positive correlation with regard to leukocyte count, (p=0.091, **Figure 5C**). However, low hemoglobin level were significantly associated with higher level of pTACI (p=0.042, **Figure 5D**). In summary, pTACI seems to be directly related to detectable changes in the peripheral blood parameters. Another clinically relevant parameter for progression of malignant disease is the Body mass index (BMI). However, no association of pTACI level or platelet activation (CD62P expression) and BMI was observed (**Figures 5E, F**). Notably, patients with higher BMI tended to be more likely to have metastasis (BMI in patients with metastasis: 26.8 ± 4.2 vs. 24.2 ± 5.3 in patients without metastasis, p=0.1).

**Figure 6:**
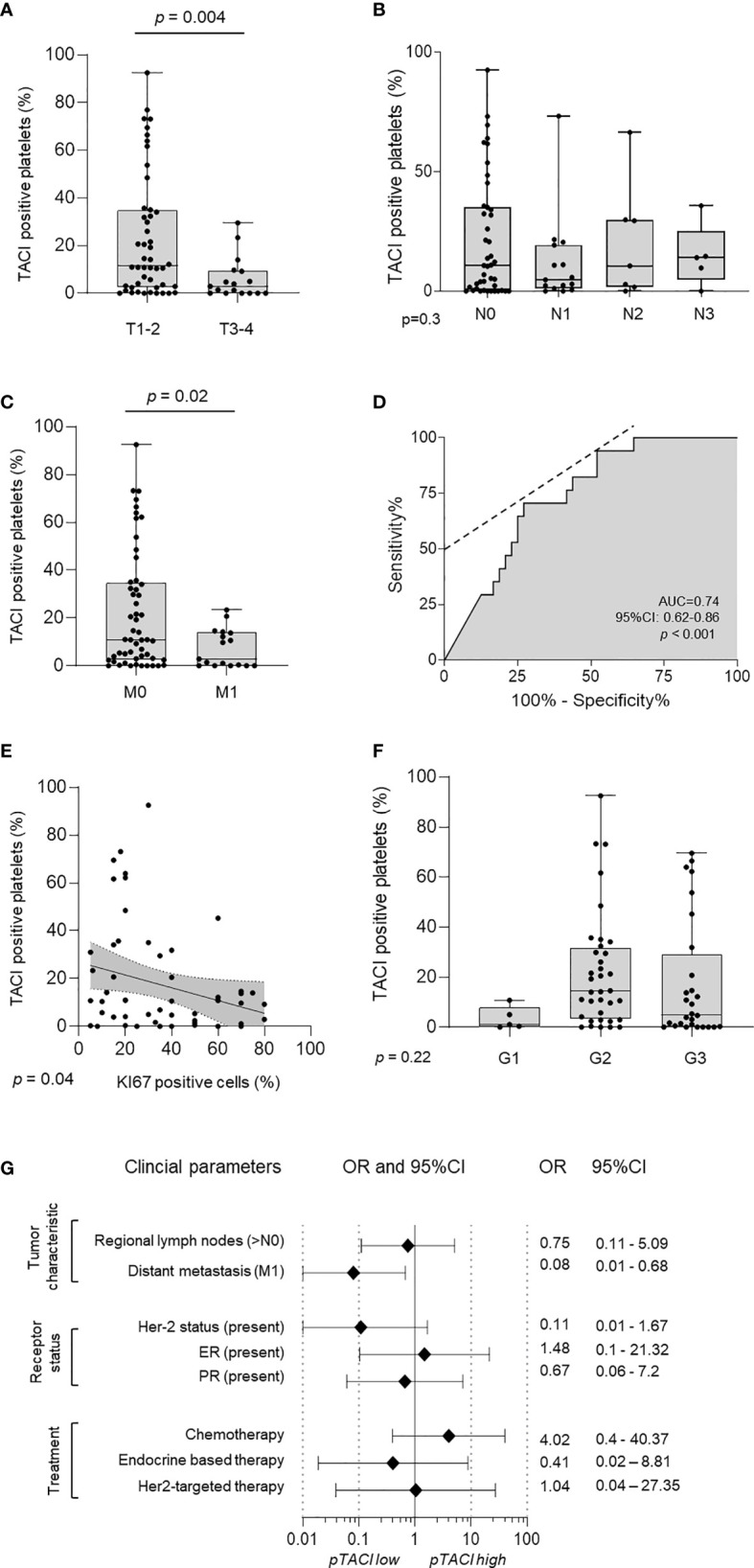
Association of pTACI expression with prognostic parameters in breast cancer. **(A–C)** Expression of pTACI according to breast cancer tumor stages (T1-2, T3-4, p = 0.004) **(A)**, lymph node invasion (N0-3, p = 0.3) **(B)** or occurrence of metastasis (M0-1, p = 0.02) **(C)**. **(D)** The predictive value of pTACI expression for metastasis was analyzed using ROC (Area under the ROC curve, AUC 0.74, 95%CI: 0.62-0.86, p < 0.001). **(E, F)** Correlation of pTACI level and tumor proliferation (% Ki67 positive tumor cells) (p = 0.04) **(E)** and different tumor grades (G1-3, p = 0.22) **(F)**. **(G)** Odds ratios (OR) of several clinical parameters and their association with pTACI level are shown. pTACI high was defined as follows: mean pTACI (HD) + 2SD pTACI (HD).

Our observation that pTACI levels on platelets from breast cancer patients were significantly increased compared to HD (**Figure 2E**) may suggest an association of pTACI with the pathophysiology of breast cancer. In a final step, we studied the relationship of pTACI expression and several clinical prognostic parameters in our breast cancer cohort. pTACI level were found to be inversely associated with tumor stages (T), as patients with lower tumor stages (T1-2) displayed significantly higher pTACI level compared to patients with higher tumor stages (T3-4), (p=0.004, **Figure 6A**).

In contrast, there was no significant association between pTACI expression and lymph node invasion (N0-N3) in the respective patients (p=0.3, **Figure 6B**). Interestingly, patients with diagnosis of metastasis (M1) displayed significantly less surface TACI on their platelets as compared to non-metastasized patients (M0) (p=0.02, **Figure 6C**). Consistent with our findings, that high tumor stages and the occurrence of metastasis were correlated with lower expression of pTACI, Ki67 expression as surrogate parameter for tumor proliferation was negatively correlated with pTACI too (p=0.04, **Figure 6E**). Compared to patients with histological grade G1 and G3 tumors, patients displaying histological G2 tumors tended to express higher pTACI level. However, this trend was not statistically significant (p=0.22, **Figure 6F**).

To further evaluate the predictive role of pTACI in breast cancer metastasis which is ultimately associated with limited treatment options and worse outcome, we performed receiver-operating characteristic (ROC) analysis. The calculated AUC was 0.74 (95%CI: 0.62 – 0.86, p<0.001)(**Figure 6D**). The specificity of pTACI to predict metastasis in our breast cancer cohort was 87.2% (95%CI: 72.6-95.7%). With an additional positive predictive value (PPV) of 70.6% (95%CI: 48.9-85.7%), pTACI proved to be a promising candidate for future biomarker studies in breast cancer.

Finally, we calculated the pTACI dependent OR for multiple tumor characteristics (**Figure 6G**). In this comparative analysis, again distant metastasis (OR 0.08) correlated with low pTACI level. Interestingly, patients displaying low pTACI level showed Her-2 overexpression (OR 0.11) and were also more likely to receive endocrine-based therapy (OR 0.41). Patients with high pTACI were more likely treated with classical chemotherapeutic agents (OR 4.02). Albeit, the expression of pTACI in patients treated with chemotherapeutic regimen tended to be higher, no statistically significant differences were obtained (p=0.09) (**Supplementary Figure 5**).

## Discussion

TACI is a receptor for APRIL, BAFF and interacts with CAML (Calcium signal-modulating cyclophilin ligand) and as such mainly known for its role in B cell immunity (26, 32). Beyond that, several TNFRSF members including TACI were found to be associated with tumor progression. Although the TME has been discussed as a major source of TNFRSF members, several studies reveal that tumor cells themselves appear to express members of this molecule family (13, 33, 34). Especially in breast cancer APRIL and BAFF were shown to promote tumor cell proliferation and induction of breast cancer stem cell signatures *via* BCMA-JNK signaling (10, 11).

We here analyzed mRNA gene expression of the receptors *BAFF-R*, *TACI* and *BCMA* in tumor biopsies of 1,006 breast cancer patients and their association with patients’ survival. The finding that high levels of the immunoregulatory TNFRSF member TACI, but not the other receptors correlated with beneficial outcome, appears to be counterintuitive at the first sight. However, bearing in mind that low TACI expression in myeloma cells was associated with a higher expression of several cell cycle genes; *TACI* gene signatures in breast cancer might reflect the dependency of the tumor cells on soluble factors, like APRIL, BAFF (35). Following this assumption, low levels of *TACI* mRNA in the TCGA breast cancer cohort might reflect a subgroup of highly proliferative tumors, independent from growth factors derived from the TME.

Since the TME and platelets in particular are increasingly appreciated to play a critical role in solid tumors, especially due to their close interaction during metastatic dissemination (36), we thus determined the corresponding expression of BAFF-R, BCMA and TACI on the surface of platelets. With this we not only describe, to our knowledge for the first time, expression of all three TNFRSF on human platelets, but beyond that observed substantially enhanced levels of pTACI - but not the other TNFRSF members - in breast cancer patients. This confirms and extends current knowledge on the expression and specific regulation of TNF(R)SF members on platelets, which is so far mainly focused on the role of platelet-derived ligands, e.g. RANKL, GITRL, CD40L, FasL, TRAIL (17, 20, 37–40). Expression/modulation of platelet proteins in general and of pTACI in particular may be a result of (a) (altered) translation during megakaryopoiesis, (b) trogocytosis which has been reported upon tumor cell/platelet interaction (41) or (c) *de novo* protein biosynthesis in platelets, which can among others occur from ingested, tumor-derived mRNA (25).

As the level of platelet surface molecules is critically dependent upon the platelet activation status and maturation (38, 39, 42) we further went on to study pTACI regulation with regards to these parameters. Of note, higher levels of pTACI were observed in resting platelets; while upon activation which among others occurs upon their interaction with tumor cells, platelets appear to specifically loose expression of the receptor. To assess, whether the regulation pTACI particularly in patients may be due to a differential activation status between both, HD and breast cancer patients, we next determined the endogenous activation (i.e. P-Selectin/CD62P expression) of platelets which however showed comparable levels in both groups. Albeit there are reports on a hyperactive platelet state in the malignant setting (43), we did not observe this phenotype when assessing CD62P level in our collective. However, we did not comprehensively study platelet functionality in our entire cohort. Moreover, it can be assumed that not all patients in our collective harbor circulating tumor cells which would upon encounter with platelets in the blood stream lead to activation of the latter. Remarkably, the level of CD62P positive platelets clearly correlated with the extent of TACI modulation in a given donor. Naturally, high pTACI surface expression was accompanied by a big change, i.e. reduction, in TACI level upon platelet activation, which was again true for both, HD and breast cancer patients. We set out to assess the underlying mechanism of pTACI downregulation. Since TACI is known to be shed by ADAM10, we assessed pTACI level of resting or TRAP-activated platelets in the presence of a broad spectrum matrix metalloprotease inhibitor or ADAM10 inhibitor. Together, these data indicate that proteolytic cleavage of pTACI is at least in part responsible for pTACI downregulation in activated platelets. Platelets express ADAM10 which may modulate several pathways in the TME (29). The question whether ADAM10 - and in particular platelet-derived ADAM10 - is relevant for pTACI shedding *in vivo* needs to be further investigated.

To further evaluate if pTACI has a functional relevance in HD and breast cancer, we assessed whether stimulation of pTACI does alter platelet function. Interestingly, presence of low or high oligomeric BAFF did neither alter platelet adhesion or platelet aggregation. Together, these data suggest that pTACI-dependent signaling alone and in combination with collagen and ADP has no influence on platelet functionality in our *in vitro* setting. Interestingly, similar observation has already been made for GITRL-dependent signaling in platelets (20). However, the situation in the presence of tumor cells *in vivo* and in particular within the TME is much more complex and further work is definitely warranted.

In order to address whether altered levels in pTACI may be associated with hematopoiesis in the respective breast cancer patients, we analyzed its association with various hematological/clinical parameters. We observed a positive correlation of pTACI level with platelet volume while it was inversely correlated with hemoglobin demonstrating that pTACI reflects alterations in the patients’ blood count. Whereas the negative correlation with hemoglobin level might reflect tumor treatment or tumor anemia, the correlation of pTACI with larger platelet volumes in breast cancer may indicate a close relationship between platelet homeostasis and cancer (44). While certainly further work is required to unravel the mechanisms underlying these relationships, processes including i) trogocytosis, i.e. transfer or membrane molecules between cells or in this case platelet and tumor cells (41), ii) transfer or RNAs between platelets and tumor cells and iii) reprogrammed megakaryopoiesis in patients (45) may contribute to the observed phenomena. Additionally, the clinical parameter BMI which was found to be negatively associated with overall survival in a meta-analysis of >210,000 breast cancer patients (46) did not correlate with pTACI level or platelet activation in our study. Of note, TACI is expressed on macrophages where it appears to mediate weight gain and a dysregulated glucose homeostasis in mice (47), and on adipocytes where it is involved in maturation (48). As pTACI may reflect TACI expression of the tumor, TME or immune cells, and it is thus tempting to speculate about putative associations in this context; however further investigation in a bigger cohort will be necessary to fully address this question.

Finally, we addressed the question whether pTACI level were associated with tumor characteristics in our breast cancer cohort. Remarkably, pTACI level were downregulated in advanced disease stages as reflected by the T stage, occurrence of metastasis and tumor proliferation index Ki67. We also observed a tendency of enhanced pTACI levels in patients receiving chemotherapy. Although chemotherapeutic regimen clearly influence megakaryopoiesis and often result in thrombopenia in the respective patients, further studies are necessary to understand whether these agents indeed modulate pTACI expression.

Most importantly, pTACI levels were able to predict the occurrence of metastasis which is strongly intertwined with the ultimate disease outcome with a high specificity. In line with the finding that lower pTACI levels were associated with higher metastatic burden we found that Her-2, which is known to be a negative prognostic marker, was more frequently expressed in patients with low pTACI level (49). While soluble TACI was suggested as a biomarker in primary central nervous system lymphoma (50), further studies are warranted to assess whether soluble (p)TACI level are also associated with disease progression and could together with pTACI surface level serve as biomarker in breast cancer.

Regarding the clinical outcome it is noteworthy to mention that expression of TACI on platelets in our study seems to reflect our findings regarding *TACI* mRNA data in the tumor cells in the TCGA cohort. Even if further studies are needed to revalidate these findings, this work highlights the involvement of platelets in tumor biology.

Together, we here show to our knowledge for the first time that platelets express TACI. pTACI associates with several clinical prognostic markers in breast cancer and more importantly has a high positive predictive value regarding the occurrence of metastasis which is closely connected to the loss of curative treatment options and hence a very strong prognostic determinant. However, further studies are warranted to establish a protocol which allows for rigorous detection of platelet surface molecules before its translation in clinical decision making. Since, pTACI can be easily accessed through liquid biopsies it shows potential as a promising new biomarker in breast cancer.

## Data Availability Statement

The datasets presented in this study can be found in online repositories. The names of the repository/repositories and accession number(s) can be found below: www.oncolnc.com, 2.7 The Cancer Genome Atlas Database (TCGA).

## Ethics Statement

The studies involving human participants were reviewed and approved by IRB (ethics committee of the Faculty of Medicine of the Eberhard Karls Universitaet Tuebingen) and of the University Hospital. The patients/participants provided their written informed consent to participate in this study.

## Author Contributions

CH, YZ, CT and SM and performed experiments. CH and SM analyzed data and wrote the initial draft of the manuscript. JH, AK, AH, MH, and CH acquired patient samples and analyzed patient data. KK contributed to designing the project and writing of the manuscript. SM, HS, LZ, and CH designed and supervised the study. All authors contributed to the article and approved the submitted version.

## Funding

This work was funded by the Deutsche Forschungsgemeinschaft (DFG, German Research Foundation) under Germany’s Excellence Strategy - EXC 2180 - 39090067 and DFG, project number 374031971–TRR 240/project B05. SM is supported by the Institutional Strategy of the University of Tuebingen (Deutsche Forschungsgemeinschaft, ZUK 63) and the Deutsche Forschungsgemeinschaft, MA 8774/1-1. KK is supported by the Mainz Research School of Translational Biomedicine (TransMed) of the University of Mainz. HS is funded by Deutsche Forschungsgemeinschaft, SA1360/7-3, Wilhelm Sander-Stiftung, 2007.115.3, and Deutsche Krebshilfe 70112914.

## Conflict of Interest

The authors declare that the research was conducted in the absence of any commercial or financial relationships that could be construed as a potential conflict of interest.
