# Supplementary material for: Platelet-Expressed TNFRSF13B (TACI) Predicts Breast Cancer Progression
Source: Front Oncol. 2021 Mar 17;11:642170. doi: 10.3389/fonc.2021.642170 (PMC8010255; doi:10.3389/fonc.2021.642170)
Supplement: Supplementary file 1 [file DataSheet_1.docx]

Supplementary Material

# Supplementary Figures

**Supplementary Figure 1.** TACI is predominantly expressed as short isoform (isoform-ID: uc002gqt.1, coding exon count: 4) which lacks the cysteine rich domain 1 and was detected in 75 % of all patients in the TCGA cohorts. The full length receptor (isoform-ID: uc002gqs.1, coding exon count: 5) was detected in 25 % of all patients; revealed by the TPM (transcripts per kilobase million) expression level.

**Supplementary Figure 2.** Platelet activation (CD62P level) correlates with pTACI expression change (Δ TACI, defined as ‘%TACI expression in endogenously activated, CD62P positive platelets’- ‘%TACI expression in CD62P negative platelets’) in breast cancer patients but not in HD (A,C).pTACI level are directly associated with pTACI downregulation in endogenously activated platelets as compared to resting platelets (B,D). Line indicates linear regression, gray area 95%CI interval.


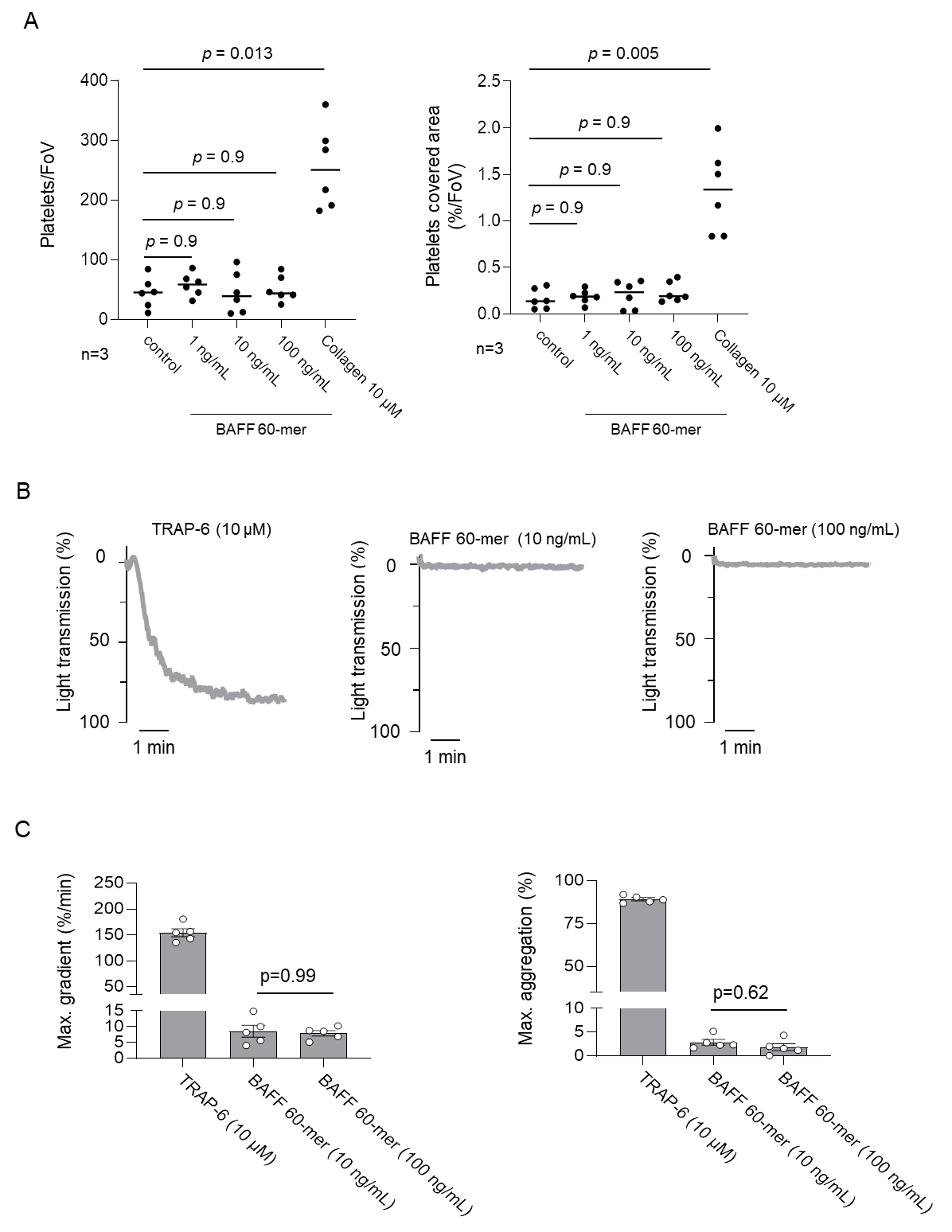


**Supplementary Figure 3. (A)** Presence of an oligomerized BAFF 60-mer did not alter platelet adhesion as determined by the number of platelets per field of view (FoV) (left panel) nor the area covered by platelets (%/FoV) (right panel). **(B-C)** BAFF 60-mer did also not induce platelet aggregation as determined by % light transmission **(B)** nor maximal aggregation **(C)**.


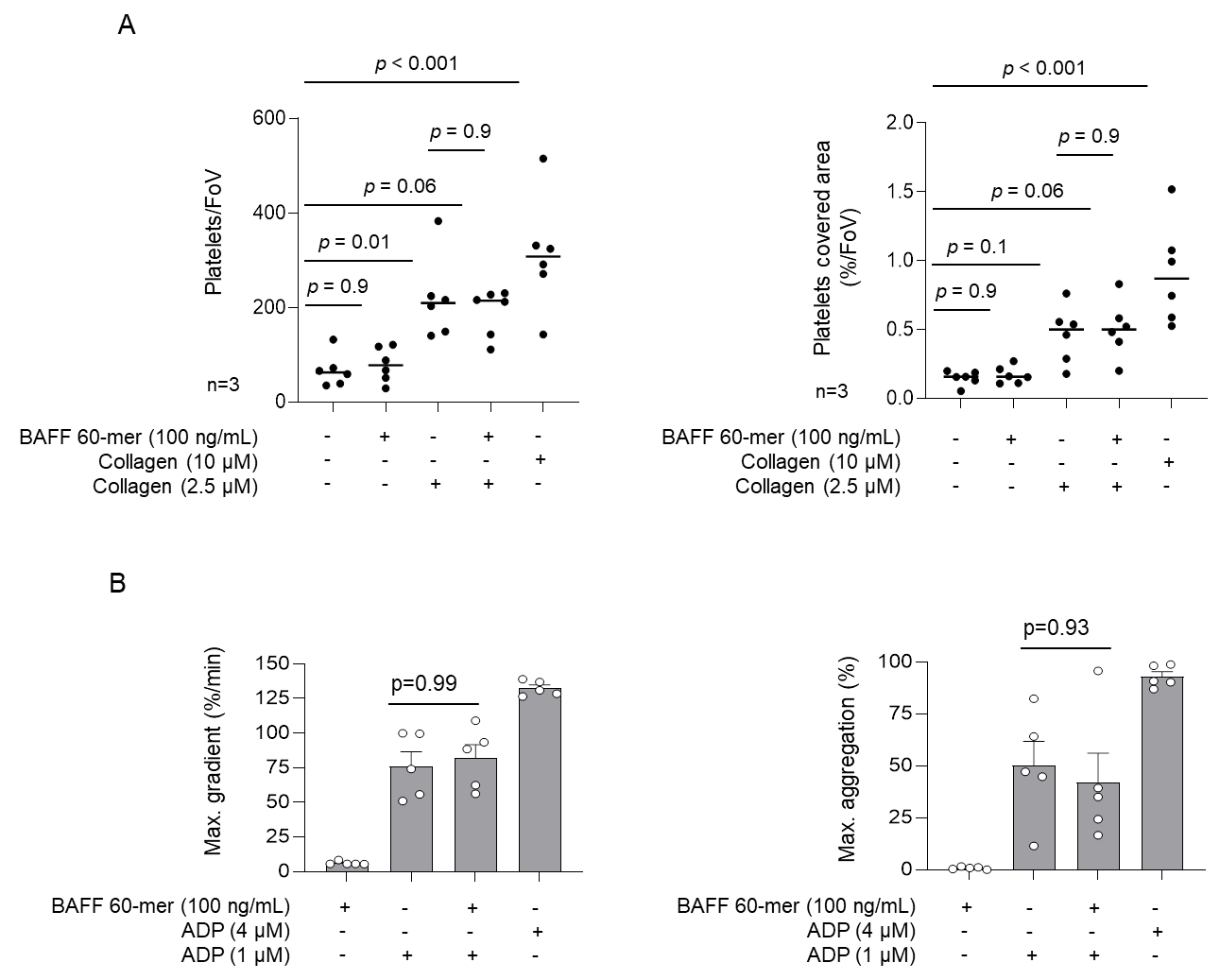


**Supplementary Figure 4. (A)** Presence of oligomerized BAFF 60-mer did not enhance platelet adhesion to collagen as determined by the number of platelets per field of view (FoV) (left panel) nor the area covered by platelets (%/FoV) (right panel) in our *ex vivo* setting. **(B)** BAFF 60-mer did not influence platelet aggregation in the presence of ADP as determined by % light transmission and maximal aggregation.


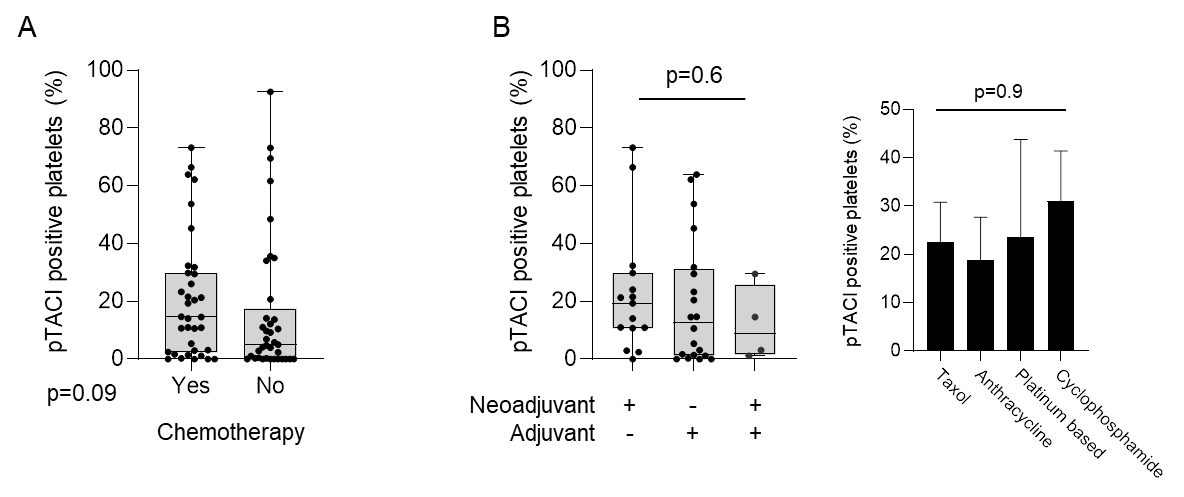


**Supplementary Figure 5. (A)** pTACI level tended to be higher in patients which received chemotherapeutic treatment (p=0.09). **(B)** There were no significant differences regarding, neoadjuvant vs. adjuvant treatment (p=0.6) or different chemotherapeutic agents (p=0.9).
